# Effect on Local Structure and Phase Transition of Perovskite-Type [N(CH_3_)_4_]_2_Zn_1-*x*_Cu_*x*_Br_4_ (*x* = 0, 0.5, 0.7, and 1) Crystals with the Various Doping of Cu^2+^ Ions

**DOI:** 10.1038/s41598-018-32022-z

**Published:** 2018-09-20

**Authors:** Ae Ran Lim

**Affiliations:** 1 Analytical Laboratory of Advanced Ferroelectric Crystals, Jeonju University, Jeonju, 55069 South Korea; 2 Department of Science Education, Jeonju University, Jeonju, 55069 South Korea

## Abstract

This study focused on how the local structures in pure [N(CH_3_)_4_]_2_ZnBr_4_ crystal are affected by the partial replacement of Zn^2+^ ions with Cu^2+^ ions. The structures and phase transition temperatures T_C_ of perovskite-type [N(CH_3_)_4_]_2_Zn_1-*x*_Cu_*x*_Br_4_ (*x* = 0, 0.5, 0.7, and 1) mixed crystals were almost unchanged by the partial doping of Cu^2+^ ions. The environments for the local structures of [N(CH_3_)_4_]_2_Zn_1-*x*_Cu_*x*_Br_4_ mixed systems were studied according to differences in the chemical shifts of the ^1^H magic angle spinning (MAS) NMR, ^13^C cross-polarization (CP)/MAS NMR, and ^14^N NMR spectra. The ^1^H and ^13^C NMR results showed that the local environments of ^1^H and ^13^C nuclei near T_C_ are not affected by substituting Zn^2+^ ions with Cu^2+^ ions, whereas the ^14^N NMR results showed that the local environment is affected near T_C_. Consequently, the main indicators of the phase transition in [N(CH_3_)_4_]_2_Zn_1-*x*_Cu_*x*_Br_4_ are related to the ferroelastic characteristics with different orientations.

## Introduction

**Figure 1:**
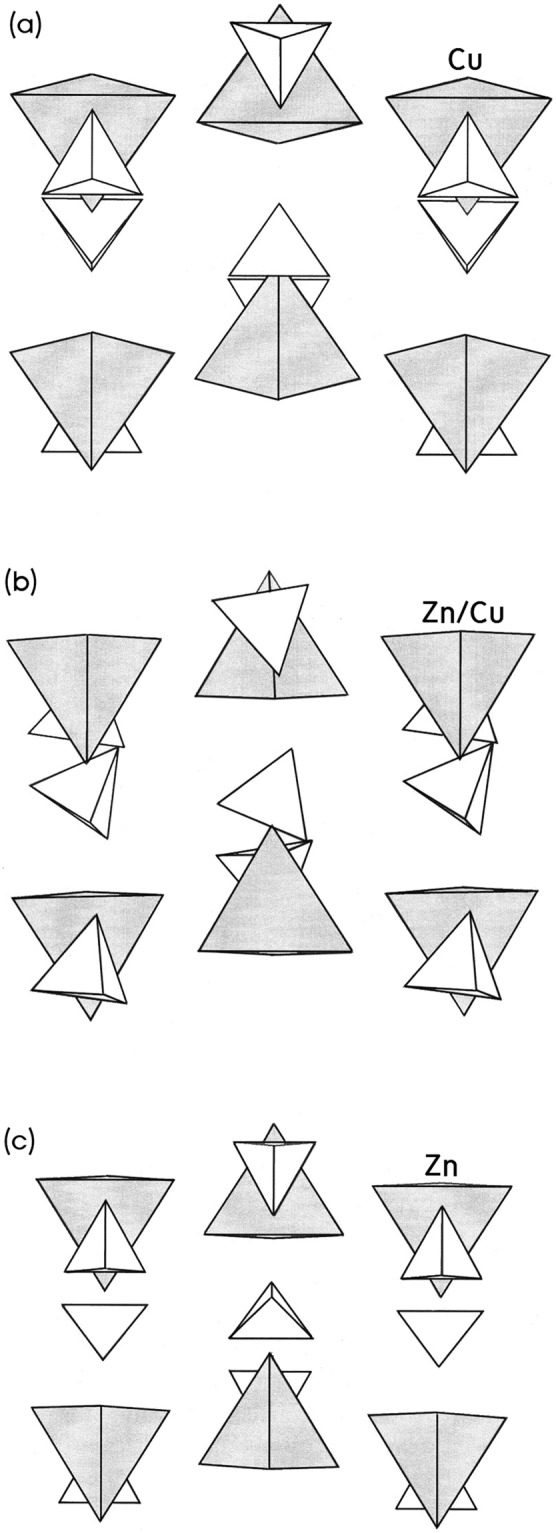
Structure of [N(CH_3_)_4_]_2_Zn_1-*x*_Cu_*x*_Br_4_ on the bc plane. Cu/ZnBr_4_^2−^ anions are represented by gray tetrahedrons. N(CH_3_)_4_^+^ cations are represented by empty tetrahedrons. (**a**) [N(CH_3_)_4_]_2_ZnBr_4_, (**b**) [N(CH_3_)_4_]_2_Zn_0.5_Cu_0.5_Br_4_, and (**c**) N(CH_3_)_4_]_2_CuBr_4._

Metal-organic hybrids, which consist of organic and inorganic components, have recently attracted much attention because these materials have many possibilities for the tailoring of their functionalities and physical properties including optical, electrical and magnetic properties. Hybrid organic-inorganic compounds based on perovskite structures are an interesting class of materials^1,2^. [N(CH_3_)_4_]_2_ZnBr_4_ and [N(CH_3_)_4_]_2_CuBr_4_ are members of the [N(CH_3_)_4_]_2_*MX*_4_ (*M* = transition metal ion; Co, Cu, Zn, Cd, and *X* = halide; Br, Cl) family. These structures undergo successive structural phase transitions, including an incommensurate–commensurate phase transition^3–11^. In the case of [N(CH_3_)_4_]_2_ZnBr_4_, the paraelastic orthorhombic phase at the phase transition temperature T_C_ = 287.6 K undergoes a second-order transition to the ferroelastic monoclinic phase^3,5,6^. The paraelastic and ferroelastic phases are denoted as I and II in order of decreasing temperature. In phase I, [N(CH_3_)_4_]_2_ZnBr_4_ has an orthorhombic structure with the space group *Pmcn* in the paraelastic phase. Its orthorhombic lattice constants are a = 12.681 Å, b = 9.239 Å, c = 16.025 Å, and Z = 4^12^. In phase II, [N(CH_3_)_4_]_2_ZnBr_4_ has a monoclinic structure with the space group *P*1*2*_*1*_*/c1*, and the lattice constants are a = 12.534 Å, b = 9.142 Å, c = 15.772 Å, γ = 89.69°, and Z = 4^13^. On the other hand, [N(CH_3_)_4_]_2_CuBr_4_ undergoes three phase transitions at 272 K (=T_C1_), 242 K (=T_C2_), and 237 K (=T_C3_) as it gradually cools^14^. The four phases are denoted as I, II, III, and IV in order of decreasing temperature. At room temperature, the crystal is in the orthorhombic phase I. As the temperature decreases, the crystal transforms to the intermediate phase II at about 272 K and then to the ferroelectric orthorhombic phase III at about 242 K. The ferroelectric phase III transforms to the lowest-temperature ferroelastic monoclinic phase IV at about 237 K^15,16^. With decreasing temperature, the crystal structure of each phase becomes orthorhombic with space group *Pnma*, incommensurate, orthorhombic with space group *Pbc*2_1_, and finally monoclinic with space group *P1*2_1_*/c1*^17–19^. At room temperature, [N(CH_3_)_4_]_2_CuBr_4_ has an orthorhombic structure with the lattice constants a = 12.600 Å, b = 9.326 Å, c = 15.825 Å, and Z = 4^20^. For two crystals, the unit cell at room temperature contains four formula units consisting of two crystallographically independent N(CH_3_)_4_^+^ ions and an *M*Br_4_^2−^ (*M* = Zn, Cu) ion. The *M*Br_4_ tetrahedron is almost undistorted, while the N(CH_3_)_4_ tetrahedra have large distortions. Figure 1 shows the crystal structures of [N(CH_3_)_4_]_2_ZnBr_4_, [N(CH_3_)_4_]_2_Zn_0.5_Cu_0.5_Br_4_, and [N(CH_3_)_4_]_2_CuBr_4_ at room temperature. The two compounds of [N(CH_3_)_4_]_2_ZnBr_4_ and [N(CH_3_)_4_]_2_CuBr_4_ have the ferroelastic property at low temperatures.

Until now, various experimental techniques have been used to report the crystal structure, phase transitions, and ferroelectricity of [N(CH_3_)_4_]_2_ZnBr_4_ and [N(CH_3_)_4_]_2_CuBr_4_^3–5,14–18^. Perret *et al*.^19^ used ^79^Br nuclear quadrupole resonance (NQR) to measure the second-order phase transition between the orthorhombic and monoclinic structures in [N(CH_3_)_4_]_2_ZnBr_4_. Recently, static nuclear magnetic resonance (NMR) and magic angle spinning (MAS) NMR spectrometry have been used to measure the chemical shifts and spin-lattice relaxation times of ^1^H and ^13^C nuclei in [N(CH_3_)_4_]_2_ZnBr_4_ as a function of temperature^20^. Two chemically inequivalent sites, N(1)(CH_3_)_4_ and N(2)(CH_3_)_4_, have been distinguished by using ^13^C cross-polarization (CP)/MAS NMR. Based on these results, the behaviors of these two chemically inequivalent N(CH_3_)_4_ groups were discussed.

In this work, perovskite-type [N(CH_3_)_4_]_2_Zn_1-*x*_Cu_*x*_Br_4_ (*x* = 0, 0.5, 0.7, and 1) mixed crystals were grown from aqueous solutions by the slow evaporation method. The ^1^H MAS NMR spectrum and ^13^C CP/MAS NMR spectrum of [N(CH_3_)_4_]_2_Zn_1-*x*_Cu_*x*_Br_4_ were measured as a function of temperature. The spin-lattice relaxation times in the rotating frame T_1ρ_ were determined for ^1^H and ^13^C nuclei in [N(CH_3_)_4_]_2_Zn_1-*x*_Cu_*x*_Br_4_ for varying amounts of paramagnetic Cu^2+^ ions. In addition, the ^14^N NMR spectrum for [N(CH_3_)_4_]_2_Zn_1-*x*_Cu_*x*_Br_4_ was observed in order to understand the role of the phase transitions. The results allowed the structural properties of pure [N(CH_3_)_4_]_2_ZnBr_4_ and [N(CH_3_)_4_]_2_CuBr_4_ to be compared, and the effect of substituting Zn^2+^ ions in [N(CH_3_)_4_]_2_ZnBr_4_ with Cu^2+^ ions was examined. And, the ferroelastic phase transition of [N(CH_3_)_4_]_2_Zn_1-*x*_Cu_*x*_Br_4_ at low temperatures was considered. This study represents the first investigation of the local structures of [N(CH_3_)_4_]_2_Zn_1-*x*_Cu_*x*_Br_4_, and the results were used to analyze the role of N(CH_3_)_4_ ions.

## Experimental Method

**Figure 2:**
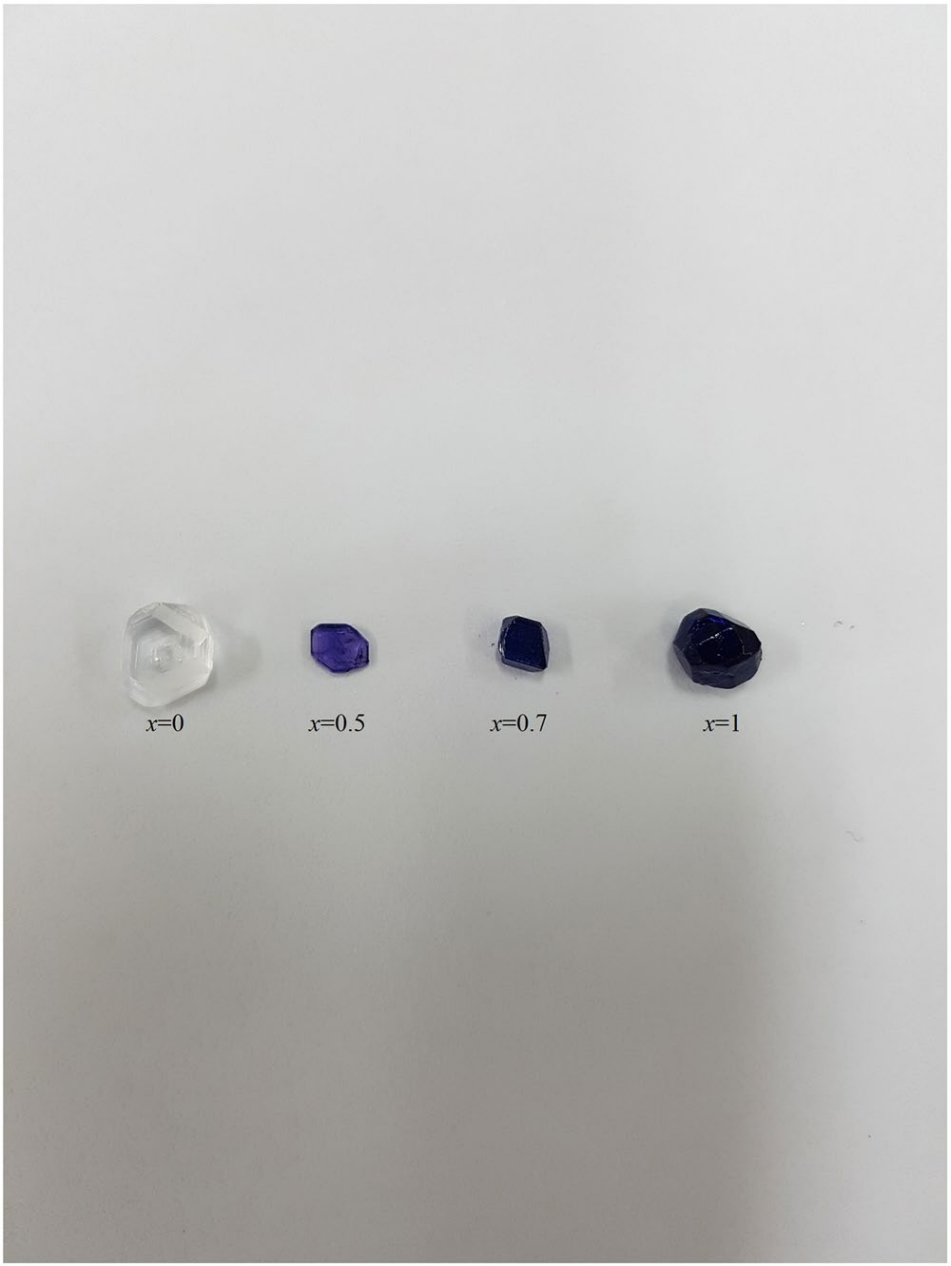
Colors of mixed crystals [N(CH_3_)_4_]_2_Zn_1-*x*_Cu_*x*_Br_4_ (*x* = 0, 0.5, 0.7, and 1).

[N(CH_3_)_4_]_2_Zn_1-*x*_Cu_*x*_Br_4_ (*x* = 0, 0.5, 0.7, and 1) single crystals were grown at room temperature by slow evaporation of an aqueous solution containing ZnBr_2_, CuBr_2_, and N(CH_3_)_4_Br in stoichiometric proportions. The [N(CH_3_)_4_]_2_Zn_1-*x*_Cu_*x*_Br_4_ single crystals varied in color according to the amount of Cu^2+^ ions, as shown in Fig. 2.

At room temperature, the structures of the [N(CH_3_)_4_]_2_Zn_1-*x*_Cu_*x*_Br_4_ (*x* = 0, 0.5, 0.7, 1) crystals were determined with an X-ray diffraction system (PANalytical, X’pert pro MPD) with a Cu–Kα (λ = 1.5418 Å) radiation source at the Korea Basic Science Institute, Western Seoul Center. Measurements were taken with θ–2θ geometry from 10° to 60° at 45 kV and with a tube power of 40 mA. Table 1 presents the lattice constants of the four crystals at room temperature. All of the [N(CH_3_)_4_]_2_Zn_1-*x*_Cu_*x*_Br_4_ crystals containing Cu^2+^ ions had the same orthorhombic structure as pure [N(CH_3_)_4_]_2_ZnBr_4_ and [N(CH_3_)_4_]_2_CuBr_4_.

**Table 1:** Lattice constants of [N(CH_3_)_4_]_2_Zn_1-*x*_Cu_*x*_Br_4_ (*x* = 0, 0.5, 0.7, and 1) at room temperature.

	*a*	*b*	*c*
[N(CH_3_)_4_]_2_ZnBr_4_ (*x* = 0)	12.691	9.244	16.012
[N(CH_3_)_4_]_2_Zn_0.5_Cu_0.5_Br_4_ (*x* = 0.5)	12.676	9.249	16.039
[N(CH_3_)_4_]_2_Zn_0.3_Cu_0.7_Br_4_ (*x* = 0.7)	12.675	9.245	16.035
[N(CH_3_)_4_]_2_CuBr_4_ (*x* = 1)	12.647	9.341	15.906

**Figure 3:**
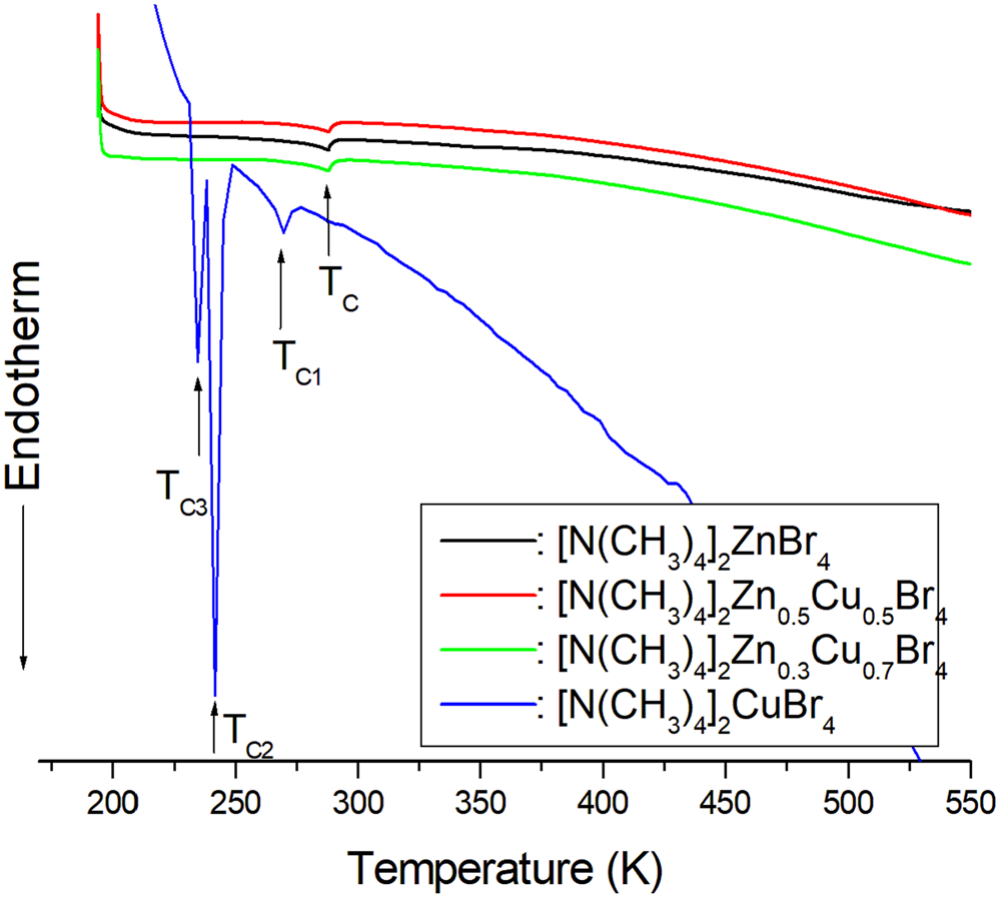
Differential scanning calorimetry (DSC) thermogram of [N(CH_3_)_4_]_2_Zn_1-*x*_Cu_*x*_Br_4_ (*x* = 0, 0.5, 0.7, and 1) single crystals.

In order to determine the phase transition temperatures, differential scanning calorimetry (DSC) was carried out on the crystals with a Dupont 2010 DSC instrument. The measurements were performed at a heating rate of 10 °C/min in the temperature range of 190–550 K. Figure 3 shows the endothermic peaks for *x* = 0, 0.5, 0.7, and 1. For [N(CH_3_)_4_]_2_Zn_1-*x*_Cu_*x*_Br_4_ (*x* = 0, 0.5, and 0.7), the DSC measurements showed only one endothermic peak at 287 K, and the phase transition temperature hardly changed when the amount of impurity Cu^2+^ ions was varied. The three endothermic peaks at 237 K (T_C3_), 245 K (T_C2_), and 272 K (T_C1_) for [N(CH_3_)_4_]_2_CuBr_4_ are related to phase transitions, and these temperatures are consistent with those previously reported^1^. When the amount of paramagnetic Cu^2+^ ions was varied, the phase transition temperatures for *x* = 0.5 and 0.7 were nearly unchanged and were similar to those for pure [N(CH_3_)_4_]_2_ZnBr_4_, although the colors of the samples were different. Thus, the impurity Cu^2+^ ions had an insignificant effect on the phase transition temperature.

The ^1^H MAS NMR and ^13^C CP/MAS NMR spectra of [N(CH_3_)_4_]_2_Zn_1-*x*_Cu_*x*_Br_4_ (*x* = 0, 0.5, 0.7, and 1) in the rotating frame were measured by using a Bruker DSX 400 FT NMR spectrometer at the Korea Basic Science Institute, Western Seoul Center. ^1^H MAS NMR and ^13^C CP/MAS NMR experiments were performed at the Larmor frequencies of 400.12 and 100.61 MHz, respectively. The samples were placed in a 4 mm CP/MAS probe as powders. The MAS rate was set to 5 kHz for ^1^H MAS and ^13^C CP/MAS to minimize the spinning sideband overlap. The chemical shifts of the spectrum for ^1^H and ^13^C nuclei were expressed with respect to tetramethylsilane (TMS). The spin–lattice relaxation times in the rotating frame T_1ρ_ for ^1^H and ^13^C were measured by using π/2-*t*-acquisition. The T_1ρ_ values were measured by varying the length of the spin-locking pulses. The π/2 pulse widths used for T_1ρ_ were 3.85 µs for ^1^H and ^13^C; this corresponded to the frequency of the spin-locking field of 64.94 kHz.

The ^14^N NMR spectra of the [N(CH_3_)_4_]_2_Zn_1-*x*_Cu_*x*_Br_4_ (*x* = 0, 0.5, 0.7, and 1) single crystals in the laboratory frame were measured by using the Bruker DSX 400 FT NMR spectrometer and Unity INOVA 600 NMR spectrometer at the Korea Basic Science Institute, Western Seoul Center. The static magnetic fields were 9.4 and 14.1 T, and the Larmor frequency was set to ω_0_/2π = 28.90 and 43.34 MHz. The ^14^N NMR experiments were performed by using a solid-state echo sequence: 4 µs–*t*–4 µs–*t*. The samples were maintained at a constant temperature with an accuracy of ±0.5 K by controlling the nitrogen gas flow and heater current. The temperature-dependent NMR measurements were carried out in the temperature range of 180–420 K.

## Experimental Results and Analysis

### ^1^H MAS NMR in [N(CH_3_)_4_]_2_Zn_1-*x*_Cu_*x*_Br_4_ (*x* = 0, 0.5, 0.7, and 1)

**Figure 4:**
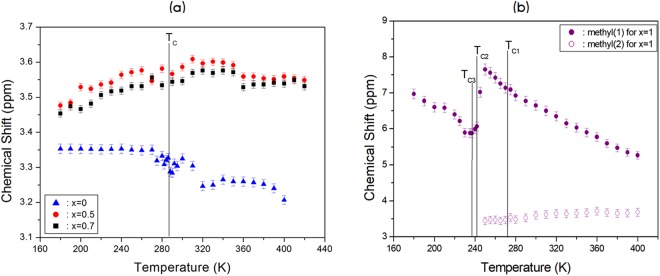
(**a**) Chemical shifts of the ^1^H MAS NMR spectrum as a function of temperature for [N(CH_3_)_4_]_2_Zn_1-*x*_Cu_*x*_Br_4_ (*x* = 0, 0.5, and 0.7). (**b**) Chemical shifts of the ^1^H MAS NMR spectrum as a function of temperature for [N(CH_3_)_4_]_2_Zn_1-*x*_Cu_*x*_Br_4_ (*x* = 1).

The ^1^H chemical shifts in order to the structural analysis of [N(CH_3_)_4_]_2_Zn_1-*x*_Cu_*x*_Br_4_ (*x* = 0, 0.5, 0.7, and 1) were carried out with the MAS NMR method. The ^1^H chemical shifts in [N(CH_3_)_4_]_2_Zn_1-*x*_Cu_*x*_Br_4_ were measured over the temperature range of 180–420 K, as shown in Fig. 4(a and b). At room temperature, the NMR spectrum of [N(CH_3_)_4_]_2_ZnBr_4_ consisted of one peak at a chemical shift of δ = 3.32 ppm, which was assigned to the methyl proton. The chemical shifts of the ^1^H NMR signal showed a slight and continuous decrease near T_C_. At *x* = 0.5 and 0.7, the chemical shifts at room temperature were 3.58 and 3.54 ppm higher, respectively, than the ^1^H chemical shift in pure [N(CH_3_)_4_]_2_ZnBr_4_. The ^1^H chemical shifts increased continuously as the temperature increased and differed from those of pure [N(CH_3_)_4_]_2_ZnBr_4_.

On the other hand, the chemical shifts at 300 K for [N(CH_3_)_4_]_2_CuBr_4_ with *x* = 1 consisted of two peaks at chemical shifts of δ = 3.60 ppm and δ = 6.65 ppm. Two chemical shifts were assigned to the methyl protons, and they may be due to two inequivalent sites of the N(CH_3_)_4_ molecule: N(1)(CH_3_)_4_ and N(2)(CH_3_)_4_. The chemical shift below T_C2_ has only one resonance line. In contrast, two resonance lines were present above T_C2_, as shown in Fig. 4(b). The chemical shifts near T_C1_ and T_C3_ were the only continuous changes, whereas there was an abrupt change near T_C2_. The change in the chemical shift indicates that a structural phase transition occurred at this temperature. The chemical shift for *x* = 1 was completely different from those for *x* = 0, 0.5, and 0.7. This difference was due to variations in the electronic structure of the Zn^2+^ and Cu^2+^ ions.

**Figure 5:**
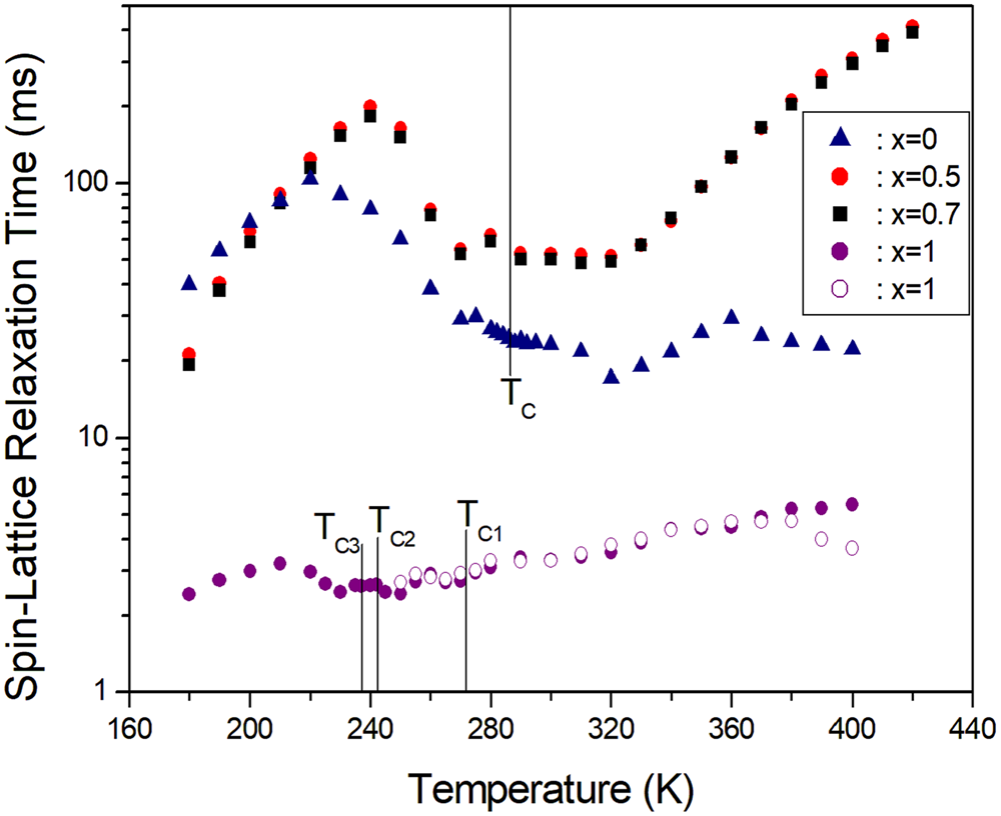
Temperature dependences of the ^1^H spin-lattice relaxation time in the rotating frame T_1ρ_ for [N(CH_3_)_4_]_2_Zn_1-*x*_Cu_*x*_Br_4_ (*x* = 0, 0.5, 0.7, and 1).

The recovery traces of the magnetization for the ^1^H nuclei in [N(CH_3_)_4_]_2_Zn_1-*x*_ Cu_*x*_Br_4_ (*x* = 0, 0.5, 0.7, and 1) were obtained at several temperatures. The saturation recovery pulse sequence was utilized to obtain the T_1ρ_ values over the whole temperature range. The nuclear magnetization recovery curves obtained for protons can be described by the following single exponential function: M(*t*) = M_0_exp(−*t*/T_1ρ_), where M(*t*) is the magnetization at the time *t*, and M_0_ is the total nuclear magnetization of ^1^H at thermal equilibrium^21^. The recovery traces of the ^1^H nuclei were measured at several delay times. Based on the slope of the plot of log M(*t*)/M_0_ versus the delay time *t*, the spin-lattice relaxation times in the rotating frame T_1ρ_ for the proton in [N(CH_3_)_4_]_2_Zn_1-*x*_Cu_*x*_Br_4_ (*x* = 0, 0.5, 0.7, and 1) were obtained as a function of the temperature, as shown in Fig. 5. When the paramagnetic Cu^2+^ ions were included, T_1ρ_ for *x* = 0.5 and 0.7 differed from ^1^H T_1ρ_ for pure [N(CH_3_)_4_]_2_ZnBr_4_, whereas the trends of ^1^H T_1ρ_ were similar with that of ^1^H T_1ρ_ for pure [N(CH_3_)_4_]_2_ZnBr_4_. ^1^H T_1ρ_ was generally continuous near T_C_. On the other hand, ^1^H T_1ρ_ of [N(CH_3_)_4_]_2_CuBr_4_ with *x* = 1 increased with the temperature. The T_1ρ_ values for N(1)(CH_3_)_4_ and N(2)(CH_3_)_4_ were nearly identical within the experimental error range. The proton T_1ρ_ data did not show any evidence of an anomalous change near the phase transition temperatures of T_C1_, T_C2_, and T_C3_. However, the ^1^H T_1ρ_ curves of [N(CH_3_)_4_]_2_Zn_0.5_Cu_0.5_Br_4_ with *x* = 0.5 and [N(CH_3_)_4_]_2_Zn_0.3_Cu_0.7_Br_4_ with *x* = 0.7 were markedly different from those observed for pure [N(CH_3_)_4_]_2_ZnBr_4_ with *x* = 0 and [N(CH_3_)_4_]_2_CuBr_4_ with *x* = 1. The ^1^H T_1ρ_ values for *x* = 0.5 and *x* = 0.7 were larger than those for [N(CH_3_)_4_]_2_ZnBr_4_ and [N(CH_3_)_4_]_2_CuBr_4._

### ^13^C CP/MAS NMR in [N(CH_3_)_4_]_2_Zn_1-x_Cu_x_Br_4_ (x = 0, 0.5, 0.7, and 1)

**Figure 6:**
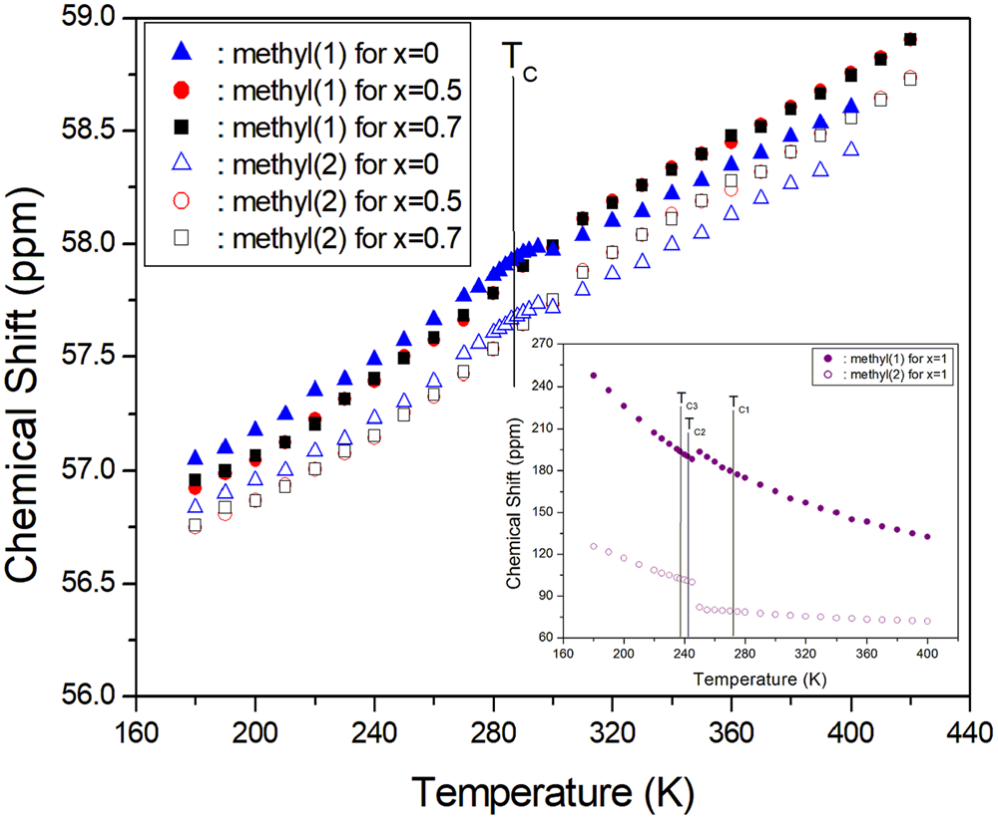
Chemical shifts of the ^13^C CP/MAS NMR spectrum as a function of temperature for [N(CH_3_)_4_]_2_Zn_1-*x*_Cu_*x*_Br_4_ (*x* = 0, 0.5, and 0.7). Inset: Chemical shifts of the ^13^C CP/MAS NMR spectrum as a function of temperature for [N(CH_3_)_4_]_2_Zn_1-*x*_Cu_*x*_Br_4_ (*x* = 1).

Structural analysis of [N(CH_3_)_4_]_2_Zn_1-*x*_Cu_*x*_Br_4_ (*x* = 0, 0.5, 0.7, and 1) was carried out with a ^13^C CP/MAS NMR method. The chemical shifts for ^13^C in [N(CH_3_)_4_]_2_Zn_1-*x*_ Cu_*x*_Cl_4_ were measured over the temperature range of 180–420 K, as shown in Fig. 6. In the case of [N(CH_3_)_4_]_2_ZnBr_4_, the ^13^C CP/MAS NMR spectrum at room temperature showed two signals at chemical shifts of δ = 57.97 and 57.72 ppm with respect to the reference TMS signal. These signals can be attributed to the methyl carbons in the two chemically inequivalent ions N(1)(CH_3_)_4_ and N(2)(CH_3_)_4_. At all temperatures, the ^13^C CP/MAS NMR spectrum of [N(CH_3_)_4_]_2_Zn_1-*x*_Cu_*x*_Br_4_ (*x* = 0, 0.5, 0.7, and 1) consisted of two resonance lines, one for N(1)(CH_3_)_4_ and the other for N(2)(CH_3_)_4_. This is shown in Fig. 6. This is because the ^13^C environments at these two chemically inequivalent sites were slightly different. The two different ^13^C resonances of N(1)(CH_3_)_4_ and N(2)(CH_3_)_4_ had almost the same chemical shift differences. This difference did not change as the temperature increased because the ^13^C environments at the two chemically inequivalent N(1)(CH_3_)_4_ and N(2)(CH_3_)_4_ changed almost equally with the temperature. The chemical shifts of the N(1)(CH_3_)_4_ ions were larger than those of the N(2)(CH_3_)_4_ ions, which is consistent with the results of previous X-ray and ^14^N NMR analyses on [N(CH_3_)_4_]_2_ZnCl_4_, which belongs to this family^22,23^. Hasebe *et al*.’s^23^ X-ray diffraction study indicated that the deformation of the N(2)(CH_3_)_4_ ion in [N(CH_3_)_4_]_2_ZnCl_4_ is larger than that of the N(1)(CH_3_)_4_ ion. Based on these results, N(1)(CH_3_)_4_ and N(2)(CH_3_)_4_ were defined according to the change in the relaxation time as a function of temperature, which was previously reported^24^. For *x* = 0.5 and 0.7, the ^13^C CP/MAS NMR spectrum of CH_3_ in the two inequivalent kinds of N(1)(CH_3_)_4_ and N(2)(CH_3_)_4_ were measured within this temperature range, and their chemical shifts were similar with that in [N(CH_3_)_4_]_2_ZnBr_4_ with *x* = 0. At 286 K, i.e., the transition temperature, the ^13^C chemical shifts for *x* = 0, 0.5, and 0.7 slowly and monotonically increased with increasing temperature. For [N(CH_3_)_4_]_2_CuBr_4_ with *x* = 1, the ^13^C CP/MAS NMR spectrum at room temperature had two signals at δ = 76.74 and 165.21 ppm. The signals at δ = 76.74 ppm and δ = 165.21 ppm represent the methyl carbons in the inequivalent N(1)(CH_3_)_4_ and N(2)(CH_3_)_4_, respectively. The chemical shifts near T_C2_ changed abruptly, whereas those near T_C3_ and T_C1_ showed a continuous change. Near T_C2_, the change in chemical shift for N(2)(CH_3_)_4_ was larger than that for N(1)(CH_3_)_4_. These results are consistent with the deformation of the N(2)(CH_3_)_4_ ion being greater than that of the N(1)(CH_3_)_4_ ion, as shown by Hasebe *et al*.’s^23^ X-ray diffraction study The ^13^C chemical shifts for *x* = 0, 0.5, and 0.7 increased with the temperature, whereas those for *x* = 1 decreased with increasing temperature. Based on these results, N(1)(CH_3_)_4_ and N(2)(CH_3_)_4_ can be defined by the change in the relaxation time as a function of the temperature. This is discussed in more detail below.

**Figure 7:**
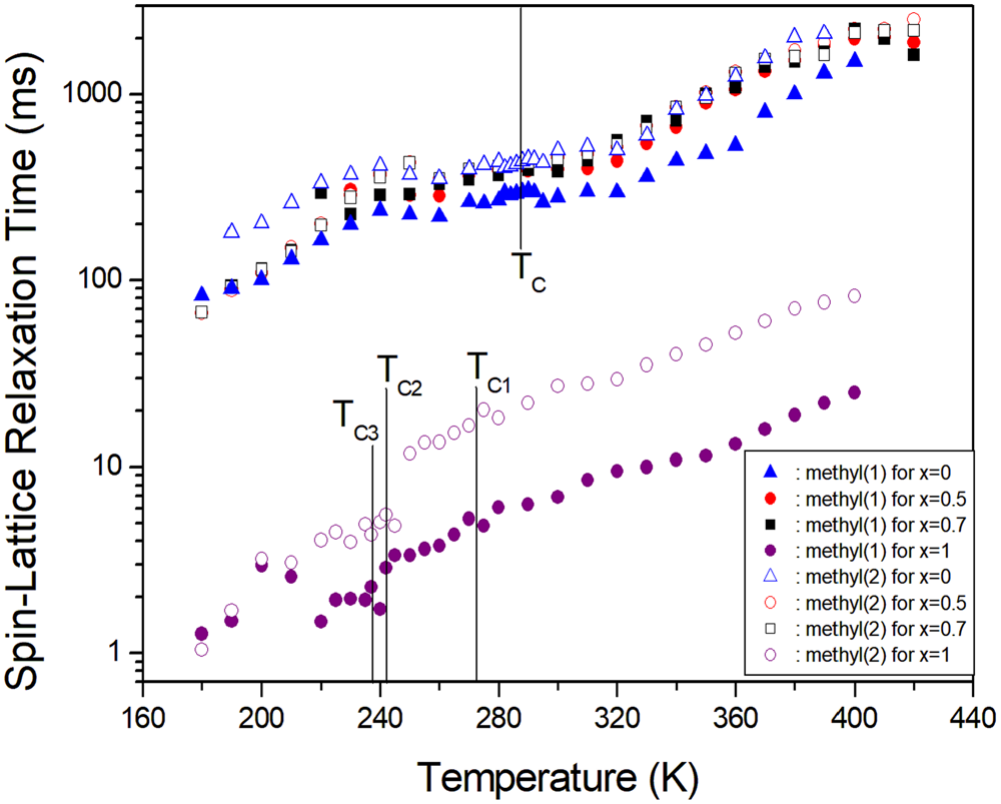
Temperature dependences of the ^13^C spin-lattice relaxation time in the rotating frame T_1ρ_ for [N(CH_3_)_4_]_2_Zn_1-*x*_Cu_*x*_Br_4_ (*x* = 0, 0.5, 0.7, and 1).

The nuclear magnetization recovery curves for carbons in [N(CH_3_)_4_]_2_Zn_1-*x*_Cu_*x*_Br_4_ (*x* = 0, 0.5, 0.7, and 1) were fitted to a single exponential function. The recovery traces of the ^13^C nuclei were measured at various delay times. Based on these results, the spin-lattice relaxation times in the rotating frame T_1ρ_ in the [N(CH_3_)_4_]_2_Zn_1-*x*_Cu_*x*_Br_4_ were obtained for each carbon as a function of temperature. Figure 7 shows the T_1ρ_ values for ^13^C in the cases of *x* = 0, 0.5, 0.7, and 1. The ^13^C T_1ρ_ values of N(1)(CH_3_)_4_ and N(2)(CH_3_)_4_ in [N(CH_3_)_4_]_2_Zn_1-*x*_Cu_*x*_Br_4_ (*x* = 0, 0.5, and 0.7) were very similar, and those for N(2)(CH_3_)_4_ were longer than those of N(1)(CH_3_)_4_. The slopes of the T_1ρ_ values near 287 K (=T_C_) were nearly continuous. In the case of [N(CH_3_)_4_]_2_CuBr_4_ with *x* = 1, the ^13^C T_1ρ_ values for N(1)(CH_3_)_4_ and N(2)(CH_3_)_4_ showed a similar trend, especially at higher temperatures. However, the change in the ^13^C T_1ρ_ value for N(2)(CH_3_)_4_ near T_C2_ was discontinuous. This result is consistent with the larger change of the ^13^C chemical shift for N(2)(CH_3_)_4_. The ^13^C T_1ρ_ values for [N(CH_3_)_4_]_2_CuBr_4_ were very small, and these T_1ρ_ values of materials containing paramagnetic Cu^2+^ ions were shorter than those of materials without paramagnetic ions. The T_1ρ_ values for CH_3_ were not affected when Zn^2+^ ions were substituted with Cu^2+^ ions in [N(CH_3_)_4_]_2_ZnBr_4_.

### ^14^N NMR in [N(CH_3_)_4_]_2_Zn_1-*x*_CuxBr_4_ (x = 0, 0.5, 0.7, and 1)

**Figure 8:**
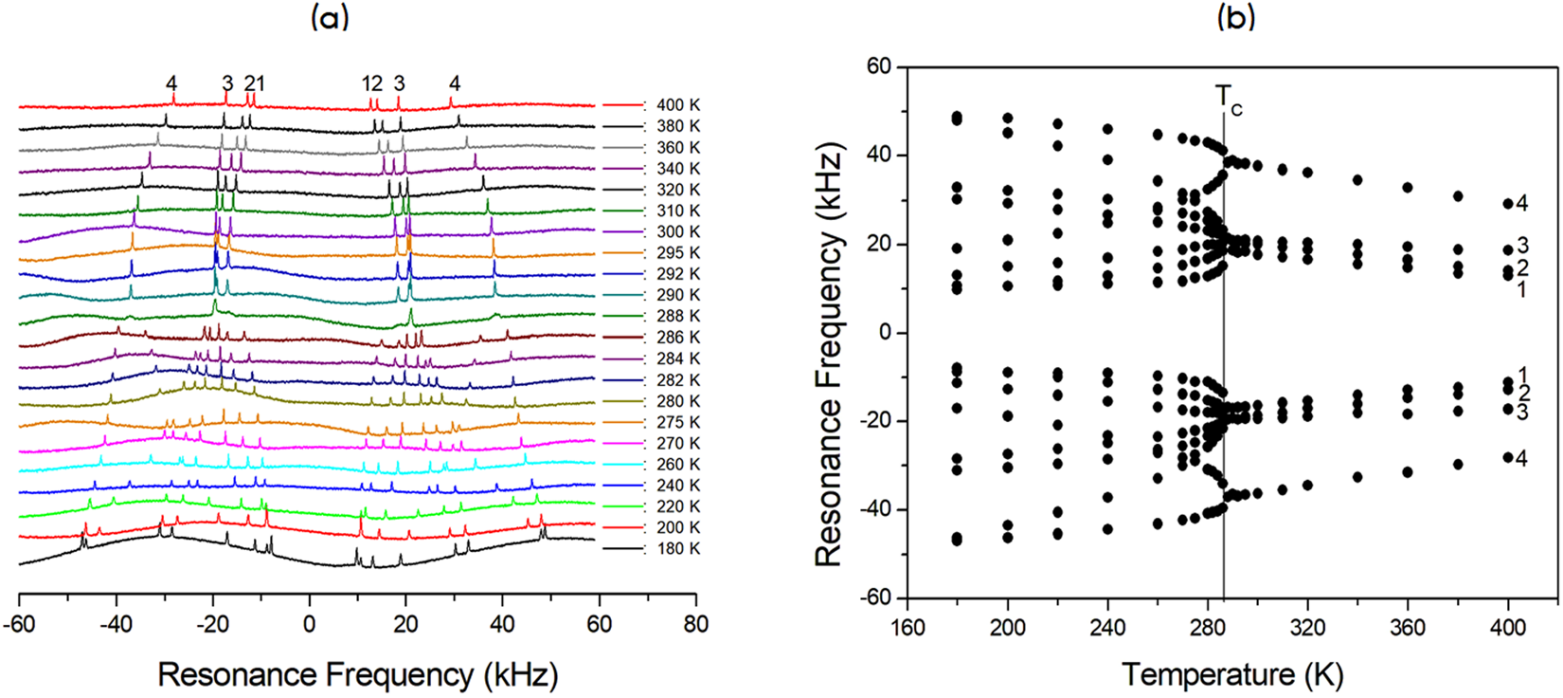
(**a**) *In-situ* ^14^N NMR spectrum as a function of temperature for [N(CH_3_)_4_]_2_Zn_1-*x*_ Cu_*x*_Br_4_ (*x* = 0). (**b**) Resonance frequency of the ^14^N NMR spectrum as a function of temperature for [N(CH_3_)_4_]_2_Zn_1-*x*_Cu_*x*_Br_4_ (*x* = 0).

**Figure 9:**
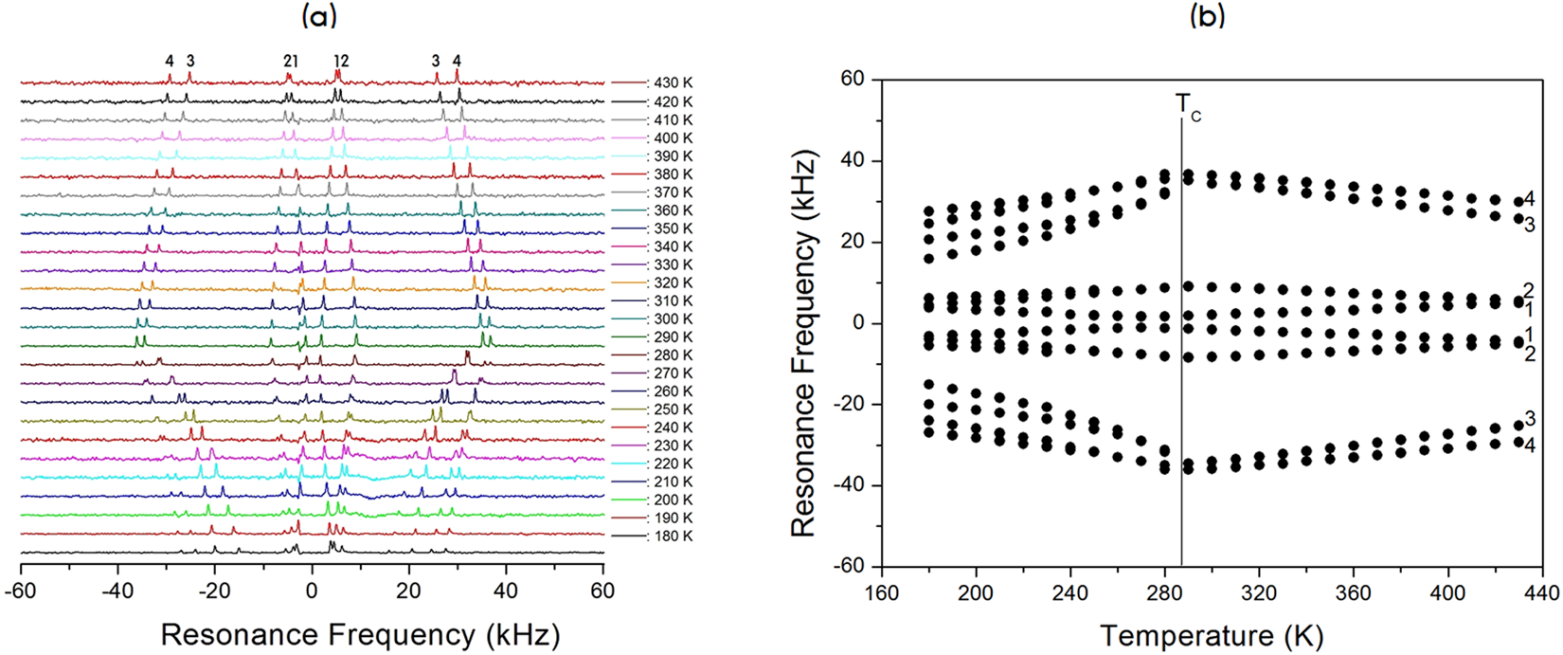
(**a**) *In-situ* ^14^N NMR spectrum as a function of temperature for [N(CH_3_)_4_]_2_Zn_1-*x*_ Cu_*x*_Br_4_ (*x* = 0.5). (**b**) Resonance frequency of the ^14^N NMR spectrum as a function of temperature for [N(CH_3_)_4_]_2_Zn_1-*x*_Cu_*x*_Br_4_ (*x* = 0.5).

**Figure 10:**
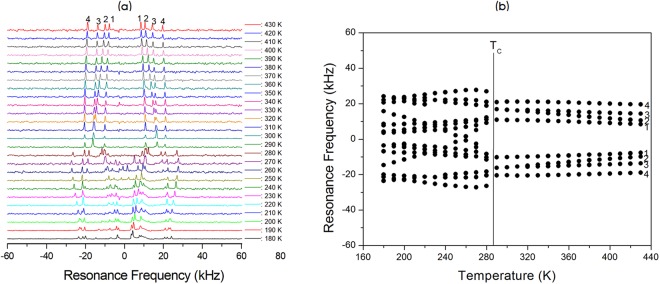
(**a**) *In-situ* ^14^N NMR spectrum as a function of temperature for [N(CH_3_)_4_]_2_Zn_1-*x*_Cu_*x*_Br_4_ (*x* = 0.7). (**b**) Resonance frequency of the ^14^N NMR spectrum as a function of temperature for [N(CH_3_)_4_]_2_Zn_1-*x*_Cu_*x*_Br_4_ (*x* = 0.7).

**Figure 11:**
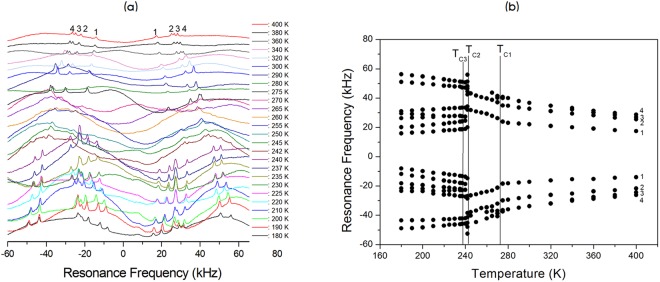
(**a**) *In-situ* ^14^N NMR spectrum as a function of temperature for [N(CH_3_)_4_]_2_Zn_1-*x*_Cu_*x*_Br_4_ (*x* = 1). (**b**) Resonance frequency of ^14^N NMR spectrum as a function of temperature for [N(CH_3_)_4_]_2_Zn_1-*x*_Cu_*x*_Br_4_ (*x* = 1).

The NMR spectra of ^14^N (I = 1) in the [N(CH_3_)_4_]_2_Zn_1-*x*_Cu_*x*_Br_4_ (*x* = 0, 0.5, 0.7, and 1) single crystal were obtained with static NMR in the laboratory frame at Larmor frequencies of ω_0_/2π = 28.90 and 43.34 MHz. Figures 8–11 show the *in situ* ^14^N NMR spectra and resonance frequencies of the ^14^N NMR spectra in the [N(CH_3_)_4_]_2_Zn_1-*x*_ Cu_*x*_Br_4_ (*x* = 0, 0.5, 0.7, and 1) single crystal as functions of the temperature. Four resonance lines for two groups at the ^14^N site in the two chemical inequivalent N(1)(CH_3_)_4_ and N(2)(CH_3_)_4_ were expected because of the quadrupole interaction of the ^14^N nucleus in [N(CH_3_)_4_]_2_Zn_1-*x*_Cu_*x*_Br_4_ single crystals. In the case of [N(CH_3_)_4_]_2_Zn_1-*x*_Cu_*x*_Br_4_ with *x* = 0, 0.5, and 0.7, the ^14^N NMR spectra above T_C_ exhibited four groups of two signals for both N(1)(CH_3_)_4_ and N(2)(CH_3_)_4_, as shown in Figs 8–10. Therefore, the eight resonance lines above T_C_ were due to two chemically inequivalent N(1)(CH_3_)_4_ and N(2)(CH_3_)_4_ ions and two magnetically inequivalent N(1)(CH_3_)_4_ and N(2)(CH_3_)_4_. This phase I–II transition resulted in an abrupt splitting of the ^14^N NMR line into several groups of lines corresponding to N(1)(CH_3_)_4_ and N(2)(CH_3_)_4_. The chemical shifts of the ^14^N signals below T_C_ varied almost continuously, and those of the ^14^N signals above this temperature also changed continuously. At low temperatures below T_C_, the ^14^N NMR signals split into 16 resonance lines. The number of resonance lines varied near the phase transition temperature, which indicates the ferroelastic twin characteristic.

On the other hand, the patterns of the resonance frequencies for [N(CH_3_)_4_]_2_CuBr_4_ with *x* = 1 changed abruptly at the phase transition temperature, as shown in Fig. 11(a and b). Between T_C2_ and T_C1_, the two lines were due to N(1) and N(2) in N(1)(CH_3_)_4_ and N(2)(CH_3_)_4_ ions, respectively. In the low-temperature region below T_C3_, the ^14^N NMR signals were split into approximately 16 resonance lines. The ^14^N NMR spectra were split into several lines for the signals arising from N(1)(CH_3_)_4_ and N(2)(CH_3_)_4_. Although the unit cell at all temperatures had Z = 4, the ^14^N resonance lines showed several resonance lines at low temperature.

Consequently, the splitting of several resonance lines near the phase transition temperatures in the [N(CH_3_)_4_]_2_Zn_1-*x*_Cu_*x*_Br_4_ indicated that a phase transition into a new phase with monoclinic symmetry occurred at this temperature, which corresponded to symmetry reduction from orthorhombic symmetry. Temperature-dependent changes in the ^14^N resonance frequency are generally due to a change in structural geometry. The electric field gradient (EFG) tensor at the N sites varied, which reflects configuration changes of atoms neighboring the ^14^N nuclei. Near the phase transition temperature, the splitting of several resonance lines of the ^14^N NMR lines for N(1)(CH_3_)_4_ and N(2)(CH_3_)_4_ were due to a ferroelastic twin domain with different orientations.

In order to confirm the ferroelastic property, the domain wall orientations were evaluated according to the spontaneous strain tensors given by Aizu^25^ and Sapriel^26^. In the transition from an orthorhombic structure with the point symmetry group *mmm* to monoclinic with the point symmetry group *2 /m*, the domain wall orientations are expressed by the following equations: *x* = 0, *z* = 0. These equations of the twin boundaries indicate the *mmm*F*2/m* ferroelastic species. During the phase transition, the point group symmetry in the crystal changed from *mmm* (phase I in case of *x* = 0, 0.5, and 0.7: phase III on case of *x* = 1) to *2/m* (phase II in case of *x* = 0, 0.5, and 0.7: phase IV in case of *x* = 1). Consequently, the NMR spectra of [N(CH_3_)_4_]_2_Zn_1-*x*_ Cu_*x*_Br_4_ (*x* = 0, 0.5, 0.7, and 1) at low temperature were attributed to the ferroelastic property, respectively.

## Discussion and Conclusion

The variation in the structural geometry as a function of the impurity concentration in the mixed system was considered according to differences in the size and electron structure between the host and impurity ions. The local structures in pure [N(CH_3_)_4_]_2_ZnBr_4_ and [N(CH_3_)_4_]_2_CuBr_4_ crystals were investigated for the effect of the random presence of a cation with a similar size. After the partial replacement of Zn^2+^ ions with Cu^2+^ ions, the Cu^2+^ ions occupied the same locations in the lattice as the Zn^2+^ ions did. The structures and phase transition temperatures of the perovskite-type [N(CH_3_)_4_]_2_Zn_1-*x*_Cu_*x*_Br_4_ (*x* = 0, 0.5, 0.7, and 1) mixed crystals were almost unchanged when [N(CH_3_)_4_]_2_ZnBr_4_ crystals were doped with Cu^2+^ ions. The environments for the local structures in [N(CH_3_)_4_]_2_Zn_1-*x*_Cu_*x*_Br_4_ (*x* = 0, 0.5, 0.7, and 1) were understood by considering the differences in chemical shifts of the ^1^H MAS NMR and ^13^C CP/MAS NMR spectra. The chemical shifts for ^1^H nuclei in [N(CH_3_)_4_]_2_Zn_1-*x*_Cu_*x*_Br_4_ varied according to the concentration of Cu^2+^ ions, whereas those for ^13^C nuclei did not change for mixed crystals with *x* = 0.5 and 0.7 when Cu^2+^ ions were added. In addition, the two crystallographically inequivalent kinds of N(1)(CH_3_)_4_ and N(2)(CH_3_)_4_ in [N(CH_3_)_4_]_2_Zn_1-*x*_Cu_*x*_Br_4_ (*x* = 0, 0.5, 0.7, and 1) were identified by using ^13^C CP/MAS NMR. The ^1^H and ^13^C spin-lattice relaxation times T_1ρ_ were obtained with varying concentrations of Cu^2+^ ions in [N(CH_3_)_4_]_2_Zn_1-*x*_Cu_*x*_Br_4_. The T_1ρ_ values for ^1^H and ^13^C nuclei were not governed by the same mechanism for a given amount of paramagnetic impurity Cu^2+^.

The roles of N(CH_3_)_4_ for the mixed systems containing the paramagnetic Cu^2+^ impurity were explained based on the ^1^H MAS NMR, ^13^C CP/MAS NMR, and ^14^N NMR data for [N(CH_3_)_4_]_2_Zn_1-*x*_Cu_*x*_Br_4_. The NMR spectra and T_1ρ_ for ^1^H and ^13^C nuclei near the phase transition temperature were not affected when Zn^2+^ ions were substituted with Cu^2+^ ions. However, the ^14^N NMR spectra were affected near the phase transition temperature. Consequently, the main indicators of the phase transition in [N(CH_3_)_4_]_2_Zn_1-*x*_Cu_*x*_Br_4_ (*x* = 0, 0.5, 0.7, and 1) were related to the ferroelastic characteristic with different orientations.
